# Increased 30-Day Emergency Department Revisits Among Homeless Patients with Mental Health Conditions

**DOI:** 10.5811/westjem.2016.6.30690

**Published:** 2016-07-26

**Authors:** Chun Nok Lam, Sanjay Arora, Michael Menchine

**Affiliations:** * University of Southern California, Department of Emergency Medicine, Los Angeles, California; † University of Southern California, USC Schaeffer Center for Health Policy and Economics, Los Angeles, California

## Abstract

**Introduction:**

Patients with mental health conditions frequently use emergency medical services. Many suffer from substance use and homelessness. If they use the emergency department (ED) as their primary source of care, potentially preventable frequent ED revisits and hospital readmissions can worsen an already crowded healthcare system. However, the magnitude to which homelessness affects health service utilization among patients with mental health conditions remains unclear in the medical community. This study assessed the impact of homelessness on 30-day ED revisits and hospital readmissions among patients presenting with mental health conditions in an urban, safety-net hospital.

**Methods:**

We conducted a secondary analysis of administrative data on all adult ED visits in 2012 in an urban safety-net hospital. Patient demographics, mental health status, homelessness, insurance coverage, level of acuity, and ED disposition per ED visit were analyzed using multilevel modeling to control for multiple visits nested within patients. We performed multivariate logistic regressions to evaluate if homelessness moderated the likelihood of mental health patients’ 30-day ED revisits and hospital readmissions.

**Results:**

Study included 139,414 adult ED visits from 92,307 unique patients (43.5±15.1 years, 51.3% male, 68.2% Hispanic/Latino). Nearly 8% of patients presented with mental health conditions, while 4.6% were homeless at any time during the study period. Among patients with mental health conditions, being homeless contributed to an additional 28.0% increase in likelihood (4.28 to 5.48 odds) of 30-day ED revisits and 38.2% increase in likelihood (2.04 to 2.82 odds) of hospital readmission, compared to non-homeless, non-mental health (NHNM) patients as the base category. Adjusted predicted probabilities showed that homeless patients presenting with mental health conditions have a 31.1% chance of returning to the ED within 30-day post discharge and a 3.7% chance of hospital readmission, compared to non-homeless patients presenting with mental health conditions (25.2%, 2.6%) and NHNM (7.7%, 1.5%).

**Conclusion:**

Homeless patients presenting with mental health conditions were more likely to return to the ED within 30 days and to be readmitted to the hospital. Interventions providing housing might improve their overall care management and have the potential to reduce ED revisits and hospital readmissions.

## INTRODUCTION

One in four American adults suffers from a mental health condition at some point.^1^,^2^ Despite a reported lower use of medical services overall,^1^,^3^ these patients have been observed to be frequent users in the emergency department (ED).^4^–^7^ Frequent ED visits could be a result of their uncontrolled health conditions.^8^–^12^ Alternatively, when they consistently use the ED as their primary source of care, the avoidable ED revisits and hospital readmissions stress an already crowded healthcare system.^10^, ^13^–^16^

Folsom et al. found that 15% of individuals with serious mental health conditions suffer from homelessness.^17^ The homeless population is vulnerable due to limited self-care ability, substance abuse and co-morbidities such as infections, skin diseases or HIV.^18^–^20^ Similar to patients with mental health conditions, many use the ED as a regular source of care.^5^, ^17^, ^21^–^23^ Studies in the U.S. and Canada have shown that providing housing to homeless individuals may result in fewer hospital days and ED visits.^18^, ^19^, ^24^–^27^

To address the medical needs of patients with mental health conditions, studies have focused on identifying factors that predict their ED revisits and hospital readmissions.^11^, ^14^ Young to middle age, male sex, Medicaid or uninsured and history of illicit drug use were among the major risk factors.^4^, ^5^, ^8^, ^20^, ^21^ However, the magnitude to which homelessness affects mental health patients’ health service utilization remains unclear in the medical community.^11^, ^28^ The objective of this study was to examine the impact of homelessness on ED revisits within 30 days post discharge and hospital readmissions among patients presenting with mental health conditions.

## METHODS

We conducted secondary analysis of administrative data in the ED at an urban safety-net hospital covering all adult ED visits in 2012. Patient age, sex, race/ethnicity, type of insurance, level of acuity, and ED disposition were obtained. We defined homelessness as having the keyword “homeless” in the address line, or if the homeless item was checked in the patient registration form. Level of acuity (Emergency Severity Index - ESI) was a five-level triage algorithm assigned at patient’s arrival, from 1 (most urgent) to 5 (least urgent). All data were de-identified. The study was approved by the institutional review board.

The study divided the ED patient population into two groups, based on whether or not the patient had presented with a mental health condition during the study period. The investigators defined patients’ mental health status by converting the primary ICD-9 at visit-level into nine categories using the validated NYU ED Algorithm using SAS 9.4 (SAS Institute, Cary NC).^16^ The NYU ED Algorithm helps classify ED utilization as being emergent, ED care needed, preventable, and whether the visits are mental health, alcohol, substance or injury related.^16^ Any patient having ≥1 visits reclassified under the “mental health principal diagnoses” category was considered as a patient ever presented with mental health conditions, regardless of the diagnoses of his/her other visits. We used this approach to minimize bias toward patient visits that were non-mental health-related that would potentially underestimate the mental health population in the patient sample.

Visit-level data and the number of visits per individual patient were recorded. The study had two outcome variables: 1) 30-day ED revisit, and 2) 30-day hospital readmission. We calculated both 30-day ED revisit and hospital readmission time based on the time difference between patients’ discharge date and the arrival date of their next visit. Any subsequent visit with a diagnosis of “aftercare” was considered a planned visit and was not captured as a revisit or readmission. The investigators extended tracking into the following year to ensure all 30-day ED revisits and hospital readmissions were captured for any ED visit taking place in December during the study period.

We used a multilevel modeling approach to account for patients with multiple visits in the dataset. Multivariate logistic regression was performed to test for moderation of homelessness on patients’ mental health status on the study outcomes. We computed adjusted predicted probabilities using regression coefficients to estimate the probability of a patient having 30-day ED revisits or hospital readmissions given his/her patient and visit characteristics. All statistical analyses used a two-sided test with α set to 0.05 and were analyzed with Stata 13.^29^

## RESULTS

The study included 139,414 adult ED visits in 2012, represented by 92,307 unique patients. Of the unique patients (43.5±15.1 years, 51.3% male, 68.2% Hispanic/Latino), 7.5% met the criteria of ever presented with mental health conditions during the study period (accounted for 11.7% of total visits), while 4.6% of patients were homeless at any time during the study period (accounted for 10.9% of total visits). Patients who presented with mental health conditions were more likely to be younger (38.3±13.6 vs. 43.9±15.1), male sex (61.1% vs. 50.5%), White (22.2% vs. 9.8%) or African-American (24.4% vs. 10.5%), and homeless (21.5% vs. 3.2%) compared to patients without mental health conditions (p<0.001). Patients who presented with mental health conditions had a higher number of total visits (2–5 visits: 35.9% vs. 21.0%, >5 visits: 6.6% vs. 1.4%), averaging 2.4±3.8 visits (range 1–184) versus 1.4±1.8 visits (range 1–98) among patients without mental health conditions (p<0.001) (Table 1 top).

In terms of visit-level data, patients who presented with mental health conditions were more likely to be covered by Medicare (9.6% vs. 4.8%, delta: 4.8%, 95% CI: [4.4, 5.2]) and other government programs (31.1% vs. 9.5%, delta: 21.6%, 95% CI: [21.1, 21.6]); to present to the ED with a lower level of acuity (ESI: 3.14 vs. 2.99, delta: 0.15, 95% CI: [0.14, 0.16]); to more frequently be transferred to outside facilities including psychiatric hospitals (33.7% vs. 4.6%, delta: 29.1%, 95% CI: [28.7, 29.6]); but less likely to be admitted to the hospital for non-psychiatric medical conditions (11.7% vs. 13.9%, delta: 2.2%, 95% CI: [1.7, 2.8]) compared to patients without mental health conditions (p<0.001). However, 42.5% of their subsequent ED visits were 30-day ED revisits, versus 17.2% by patients without mental health conditions (delta: 25.3%, 95% CI: [24.7, 26.0], p<0.001). In addition, 4.7% of their ED revisits resulted in hospital readmission, compared to 2.6% by patients without mental health conditions (delta: 2.1%, 95% CI: [1.8, 2.3], p<0.001).(Table 1 bottom).

Results from multilevel multivariate logistic regression (Table 2 top) showed a significant interaction between homelessness and mental health status. Overall, homeless patients presented with mental health conditions were more likely to have 30-day ED revisits (OR: 5.48, 95% CI: [4.85–6.18], p<0.001) and hospital readmissions (OR: 2.82, 95% CI: [2.31–3.46], p<0.001) compared to non-homeless, non-mental health (NHNM) patients. Among patients presenting with mental health conditions, being homeless contributed to an additional 28.0% increase in likelihood (4.28 to 5.48 odds) of 30-day ED revisits and 38.2% increase (2.04 to 2.82 odds) of hospital readmissions, while adjusting for other covariates. As for homeless patients, those presenting with mental health conditions contributed to an additional 181.0% increase in likelihood (1.95 to 5.48 odds) of 30-day ED revisits and a 64.0% increase (1.72 to 2.82 odds) of hospital readmissions.

Results from adjusted predicted probabilities showed that being homeless increased mental health patients’ probability of returning to the ED within 30 days from 25.2% to 31.1%. For hospital readmissions, the probability increased from 2.6% to 3.7%. Comparing to non-homeless non-mental health (NHNM), homeless patients presenting with mental health conditions demonstrated a four-fold increase (31.1% vs. 7.7%) in 30-day ED revisits, and 2.5-fold increase (3.7% vs. 1.5%) in hospital readmissions compared to NHNM patients (Table 2 bottom).

## DISCUSSION

The findings that homeless patients and those presenting with mental health conditions have a significant increase in 30-day ED revisits and hospital readmissions compared to patients with neither of these problems is mostly consistent with prior literature on these patient groups. 14–7, 17, 21–23 What is novel in our study is finding that being homeless and having a mental health condition interact to produce even higher use, which likely contributes to an already crowded healthcare system.^10^, ^13^–^16^ These findings highlight the need for ED resources to treat patients in either of these categories.

Current literature on ED revisits and hospital readmissions is limited by the lack of advanced statistical modeling to account for within-patient level data.^11^, ^14^, ^20^ Using multilevel modeling helped control for the within-patient clustering effects so that patient characteristics of the repeated visitors would not be overrepresented in the estimations. In addition, testing for the interaction between homelessness and mental health conditions provided a quantitative measure and magnitude on either category to predict the study outcomes. As suggested in the literature,^19^ the two conditions interact in limiting an individual’s ability to care for self. This results in an increased likelihood of seeking more advanced-stage medical treatments.

The study has several limitations. Homelessness was a self-report measure; institutionalized individuals who provided an address of their shelter were eligible for inclusion without additional verification. This approach is a conservative definition of homelessness and if anything would tend to underestimate the odds of recidivism. The study also focused on a single-site, urban safety-net hospital, which might limit the generalizability. To define patients’ mental health status, the investigators only used patients’ primary diagnosis and might underestimate the prevalence of ED patients with mental health conditions. If this is the case, our estimates of the odds of recidivism for the mental health and homeless population will be biased downward (e.g. the actual odds ratios would be greater than we observed). However, the study results provided an estimate of the underserved, low-income patient population that was more likely to suffer from homelessness and mental health conditions. The data are practical given they reflect on a sample living in Los Angeles, a city where the rates of both conditions rank among the top in the nation, with high demand for medical needs but limited resources to address the problems.^30^, ^31^

## CONCLUSION

In summary, homelessness increased the likelihood of 30-day ED revisit and hospital readmission among patients who presented with mental health conditions. Interventions such as providing housing may improve their overall care management and have the potential to reduce their ED revisits and hospital readmissions over time.

## Figures and Tables

**Table 1:** Characteristics of patient-level and visit-level data, by mental health status.

Characteristics	Total		Mental health		Non-mental health		p-value
		
	n	%	n	%	n	%	
Patient-level data	n=92,307		n=6,933 (7.5%)		n= 85,375 (92.5%)		

Age (mean, SD)	43.5	15.1	38.3	13.6	43.9	15.1	<0.001
Male	47311	51.3%	4237	61.1%	43074	50.5%	<0.001
Race/ethnicity							
White	9919	10.8%	1541	22.2%	8378	9.8%	
Asian	6021	6.5%	388	5.6%	5633	6.6%	<0.001
African-American	10637	11.5%	1694	24.4%	8943	10.5%	
Latino	62914	68.2%	3147	45.4%	59767	70.1%	
Other	2815	3.1%	163	2.4%	2652	3.1%	
Homeless*	4210	4.6%	1493	21.5%	2717	3.2%	<0.001
Number of total visits							
1	70,233	76.1%	3,991	57.6%	66,232	77.6%	<0.001
2–5	20,438	22.1%	2,486	35.9%	17,952	21.0%	
>5	1,646	1.8%	456	6.6%	1,190	1.4%	
mean, SD	1.5	2.0	2.4	3.8	1.4	1.8	<0.001
median, range	1	1–184	1	1–184	1	1–98	

Visit-level data	n=139,414		n=16,303 (11.7%)		n=123,111 (88.3%)		

Insurance							
Private	3,473	2.5%	274	1.7%	3,199	2.6%	
Medicare	7,424	5.3%	1,558	9.6%	5,866	4.8%	<0.001
Medicaid	50,153	36.0%	5,895	36.2%	44,258	36.0%	
Other government programs	16,797	12.1%	5,075	31.1%	11,722	9.5%	
No insurance	61,567	44.2%	3,501	21.5%	58,066	47.2%	
Homeless (per visit)	7,231	5.2%	2,784	17.1%	4,447	3.6%	<0.001
Homeless (by patients with * definition)	15,159	10.9%	5,950	36.5%	9,209	7.5%	<0.001
Level of acuity (mean, 95% CI)**	3.01	3.01–3.01	3.14	3.13–3.15	2.99	2.99–3.00	<0.001
ED disposition							
Admitted to hospital	19,068	13.7%	1,911	11.7%	17,157	13.9%	
Transferred	11,158	8.0%	5,501	33.7%	5,657	4.6%	<0.001
Against medical advice	17,818	12.8%	1,955	12.0%	15,863	12.9%	
Home	91,370	65.5%	6,936	42.5%	84,434	68.6%	
ED revisit, ≤30 days	28,080	20.1%	6,935	42.5%	21,145	17.2%	<0.001
Hospital readmission	4,010	2.9%	761	4.7%	3,249	2.6%	<0.001

* Homelessness - at any time during study period

** Level of Acuity: score 1–5, 1 referring to highest acuity

*SD*, standard deviation; *CI,* confidence interval; *ED,* emergency department

**Table 2:** Multilevel multivariate logistic regression analysis (n=92,307) showing interaction between homelessness and mental health status.

Independent variables	ED revisit, ≤30 days		Hospital readmission	
	
	Odds ratio	95% CI	Odds ratio	95% CI
MH × homeless	5.48***	4.85–6.18	2.82***	2.31–3.46
MH × non-homeless	4.28***	3.99–4.59	2.04***	1.80–2.31
Non-MH × homeless	1.95***	1.77–2.15	1.72***	1.46–2.04
Non-MH × non-homeless	ref		ref	
Age	1.01***	1.01–1.0.1	1.01***	1.01–1.02
Male	1.33***	1.27–1.38	1.27***	1.17–1.37
Race/ethnicity				
White	ref		ref	
African-American	1.39***	1.29–1.50	0.96	0.83–1.11
Latino/Hispanic	0.93*	0.87–0.99	1.07	0.95–1.21
Asian	0.77***	0.70–0.86	1.04	0.87–1.25
Other	0.27***	0.22–0.33	0.38	0.24–0.60
Insurance				
No insurance	ref		ref	
Medicare	0.76***	0.69–0.84	1.18	0.99–1.39
Medicaid	1.35***	1.29–1.41	2.60***	2.38–2.85
Other government programs	0.65***	0.61–0.70	1.13	0.99–1.30
Private	0.38***	0.32–0.44	0.55**	0.39–0.78
Level of acuity**	0.94***	0.92–0.96	0.64	0.61–0.68
ED disposition				
Home	ref		ref	
Admitted to hospital	1.03	0.97–1.09	2.12***	1.93–2.31
Transferred	0.97	0.90–1.04	1.24**	1.09–1.42
Against medical advice	1.82***	1.72–1.93	1.29***	1.13–1.45

Adjusted predicted probabilities				

MH × homeless	31.1%		3.7%	
MH × non-homeless	25.2%		2.6%	
Non-MH × homeless	15.0%		2.8%	
Non-MH × non-homeless	7.7%		1.5%	

* p<0.05,

** p<0.01,

*** p<0.001

** Level of Acuity: score 1–5, 1 referring to highest acuity

*MH,* mental health; *Ref,* reference category; *ED,* emergency department
